# Short Communication: A simple inert pump for use with concentrated acids in automatic systems

**DOI:** 10.1155/S1463924678000127

**Published:** 1978

**Authors:** R. G. Lidzey

**Affiliations:** Automatic Methods and Computing Group Laboratory of the Government Chemist Cornwall House Stamford St. London SE1 9NQ UK


In conclusion, the kits have been used as a means to evaluate the performance of this analyser. In doing so, it is important to realise that the total variance of the system has as components the errors due to the kits as well as the Chemetrics analyser.

The total system variance was well within the chosen criteria of acceptability. Therefore the performance of the analyser itself must have been acceptable.

REFERENCES

[1] Broughton, P. M. G., Gowenlock, A. H., McCormack, J. J., and Neill, D. W., (1974). A revised Scheme for the Evaluation of Automotive Instruments for use in Clinical Chemistry. *Am. Clin. Biochem,* **11,** 207.

[2] Westgard, S. O., Carey, R. N., Wold, S. (1974). Criteria for Judging Precision and Accuracy in Method Development and Evaluation. *Clin. Chem.* **20,** 825-833.

[3] Barnet, R. N. and Jouden, W. J. (1970). A revised scheme for the comparison of quantitative methods. *American Journal of Clinical Pathology,* **54,** 454-462.

[4] Logan, J. E. (1972). Evaluation of commercial kits. *C.R.C. Critical Reviews in Clinical Laboratory Sciences,* **3,** 271-289.

### Short Communication

### A simple inert pump for use with concentrated acids in automatic systems

**R. G. Lidzey**

*Automatic Methods and Computing Group, Laboratory of the Government Chemist, Cornwall House, Stamford St., London, SE1 9NQ, UK.*

**SAMPLE** pretreatment is an important part of automatic analysis and the transfer of liquids such as corrosive acids and organic solvents is frequently required. Commercially available pumps and dispensers are often not completely inert, interfering substances can be leached from components into the analytical stream and thereby affect subsequent analytical measurements. When inert pumps are available they are rarely suitable for laboratory scale automated analytical systems, either because their flow rates are far too high, or because they are very expensive. Considerable efforts have been made in this Laboratory to develop inert pumping and dispensing assemblies, particularly for use with hot concentrated sulphuric and nitric acids.

A simple pump has been used on a previous occasion in this Laboratory [1, 2] for the transfer of acid digests in trace metal analysis. A rather more flexible system is being used now for adding nitric acid to a distillation flask at a few millilitres per minute. It consists of an assembly of mainly commercial fittings and may be described as a pump or dispenser. One item which was constructed, a non-return valve shown in figure 1, was made from PTFE and Kel-F and has screwed end connections suitable for Chromatronix or Altex flanged PTFE tube couplings. Figure 2 is a diagram of the assembly and shows the arrangement used, liquid is drawn in via one non-return valve when the syringe is operated by an air cylinder which is controlled by a 5 port valve. Settings on this valve limit the rate of filling or emptying of the syringe. A timer determines the

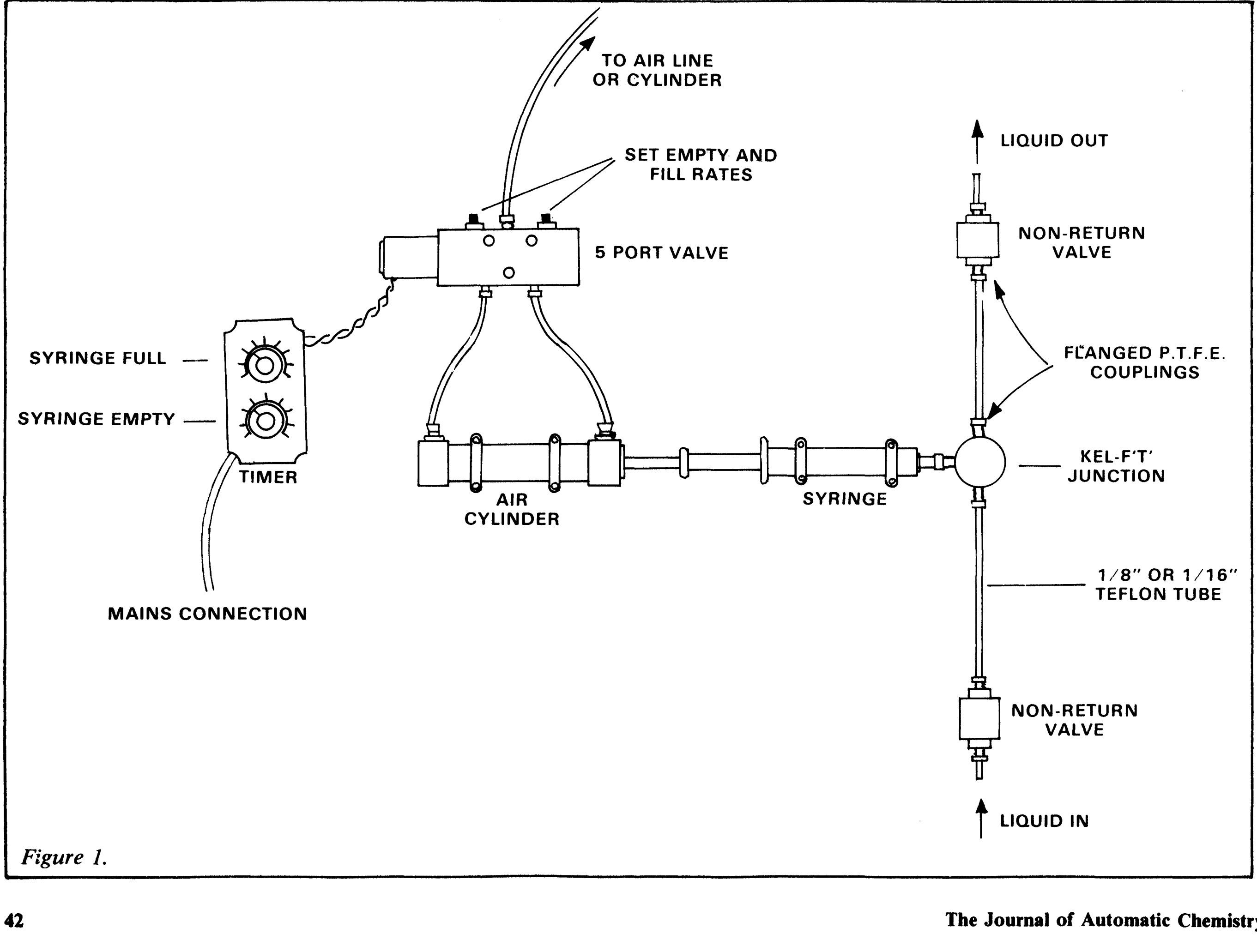

*Figure 1.*

period during which the valve is either on or off and the rate of pumping. An air supply from a cylinder or air line at a pressure of a few kilograms per square centimetre is required.

The device is very flexible, the rate of flow can be quickly adjusted by altering the timer settings or changing the syringe size or stroke and it will work for long periods without failure. It is not suitable for continuous analysis systems due to the pulsating flow but it can be used for transferring any organic or inorganic liquid at regular intervals or rates. The pressure developed can exceed 25 kilograms per square centimetre and will depend on the air line pressure.

The cost of the syringe, timer, valves and cylinder is about £80. Proprietary items were purchased from:—

Syringe — Hamilton, Gas-tight
Timer —F. R. Electronics Ltd., Cycling Timer TS-CT2C
Valve —H. Kuhnke Ltd., NWR spool valve
Cylinder —H. Kuhnke Ltd., Miniature cylinder

REFERENCES
[1] C. J. Jackson, D. G. Porter, A. L. Dennis and P. B. Stockwell (1978) *Analyst*, **103**, 317
[2] R. G. Lidzey, C. J. Jackson and D. G. Porter (1977) *Laboratory Practice*, **26**, 400

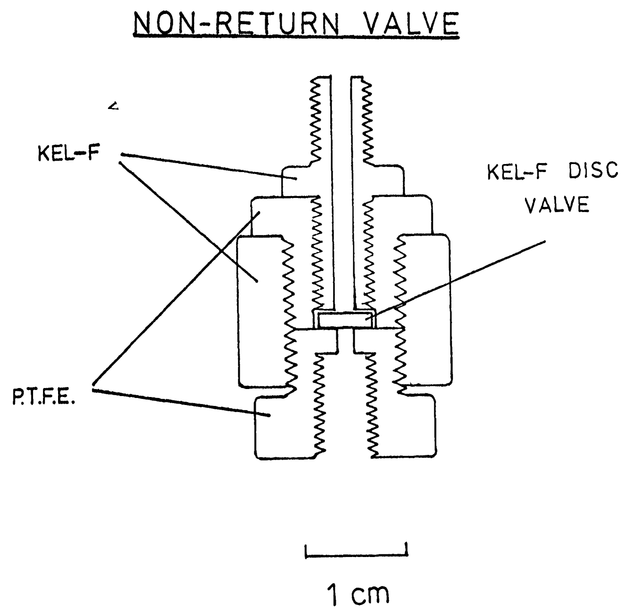

*Figure 1*

### Meeting Reports

### Chromatography Symposium

The 12th International Symposium on Chromatography was held in Baden-Baden, Germany, from the 25th-29th September 1978. The meeting was organised by the Gellschaft Deutscher Chemiker in conjunction with the Chromatography Discussion Group, Groupement pour l'Avancement des Methodes Spectroscopiques et Physiocochimiques d'Analyse, and Arbeitskreis Chromatographie der Fachgruppe Analytische Chemie. It was attended by over 700 delegates from more than 25 countries. The city of Baden-Baden, particularly the conference centre itself, was an ideal setting for such a meeting. As delegates converged on Baden-Baden they were greeted by splendid sunshine which lasted until midway through the meeting when the rain began to dampen one's spirits slightly. The former added to the warmth of the meeting and the latter no doubt helped account for the high attendance at the various scientific sessions. These were organised as a series of plenary, contributed and review lectures and these, like the weather, varied between the good and the not so good. Each lecture had previously been submitted and for the most part fully refereed to appear in a concise conference handbook produced by the Publishers Elsevier and they should be congratulated on this achievement. Such a format does, however, deviate from the original style of these symposia where the papers were available in a pre-printed form prior to the symposia and the handbook itself included any relevant discussion on the papers. This would seem more acceptable and allows speakers to concentrate on the major interest in their papers particularly on recent findings and for more detailed and fruitful discussions. This comment is, however, not meant to detract from the success that the organisers did in fact achieve — the programme catered for a wide range of tastes both with regard to the technique of chromatography and from the areas of applications these ranged from medical applications, and spirit analysis through to such problems as geochemical analysis. The meeting was however highly successful in providing a pleasant meeting place for chromatographers generally to discuss their problems with other workers and instrument companies.

The scientific content of the symposium was varied and interesting and included papers dealing with the examination of materials as diverse as geochemical substances, gin and biological fluids. Capillary column GC and GC-MS figured to a great extent, whilst less sophisticated techniques such as exclusion chromatography also received ample coverage.

Schomburg, as one has come to expect, gave an excellent review of the application of capillary gas chromatography; in this the value of two dimensional chromatographic analysis, or column switching, was indicated. Also with regard to capillary columns, there was a stark contrast between the approach of Guichon with columns over 1 km long and of the rather short columns used by Liberti. Each approach offers a number of possibilities for the analyst.

An excellent review lecture by E. Jellum (Oslo) illustrated the usefulness of capillary column GC-MS in conjunction with a spectral retrieval system, for the analysis of blood and urine samples to enable various metabolic disorders to be pinpointed. Dr. Jellum indicated that analysis was being carried out on blood given by donors over a period of years in an attempt to establish the presence of any "marker" substance that could be correlated with the onset of cancer. The implications of this study are manifest and demonstrate the potential benefits of improved chromatographic technology to the bio-sciences.

Whilst the subject of the lecture programme barely touched on the problems and applications of automation, the exhibition did include some aspects of automation and mention of some of these are made here. It is perhaps disappointing that those aspects of automation which receive most attention here are those related to microprocessor control and data processing and sample injection; the only total automation system due to be presented by Technicon did not in fact appear. It was probably most pleasing to see the increasing importance placed by instrument manufacturers on the provision of column switching techniques which significantly improve the performance specification of many separations. This is particularly true

